# Artificial intelligence and medical education: application in classroom instruction and student assessment using a pharmacology & therapeutics case study

**DOI:** 10.1186/s12909-024-05365-7

**Published:** 2024-04-22

**Authors:** Kannan Sridharan, Reginald P. Sequeira

**Affiliations:** Department of Pharmacology & Therapeutics, College of Medicine & Medical Sciences, Arabian Gulf University, Manama, Kingdom of Bahrain

**Keywords:** Medical education, Pharmacology, Therapeutics, Assessment, SLOs, OSPE, MCQs

## Abstract

**Background:**

Artificial intelligence (AI) tools are designed to create or generate content from their trained parameters using an online conversational interface. AI has opened new avenues in redefining the role boundaries of teachers and learners and has the potential to impact the teaching-learning process.

**Methods:**

In this descriptive proof-of- concept cross-sectional study we have explored the application of three generative AI tools on drug treatment of hypertension theme to generate: (1) specific learning outcomes (SLOs); (2) test items (MCQs- A type and case cluster; SAQs; OSPE); (3) test standard-setting parameters for medical students.

**Results:**

Analysis of AI-generated output showed profound homology but divergence in quality and responsiveness to refining search queries. The SLOs identified key domains of antihypertensive pharmacology and therapeutics relevant to stages of the medical program, stated with appropriate action verbs as per Bloom’s taxonomy. Test items often had clinical vignettes aligned with the key domain stated in search queries. Some test items related to A-type MCQs had construction defects, multiple correct answers, and dubious appropriateness to the learner’s stage. ChatGPT generated explanations for test items, this enhancing usefulness to support self-study by learners. Integrated case-cluster items had focused clinical case description vignettes, integration across disciplines, and targeted higher levels of competencies. The response of AI tools on standard-setting varied. Individual questions for each SAQ clinical scenario were mostly open-ended. The AI-generated OSPE test items were appropriate for the learner’s stage and identified relevant pharmacotherapeutic issues. The model answers supplied for both SAQs and OSPEs can aid course instructors in planning classroom lessons, identifying suitable instructional methods, establishing rubrics for grading, and for learners as a study guide. Key lessons learnt for improving AI-generated test item quality are outlined.

**Conclusions:**

AI tools are useful adjuncts to plan instructional methods, identify themes for test blueprinting, generate test items, and guide test standard-setting appropriate to learners’ stage in the medical program. However, experts need to review the content validity of AI-generated output. We expect AIs to influence the medical education landscape to empower learners, and to align competencies with curriculum implementation. AI literacy is an essential competency for health professionals.

**Supplementary Information:**

The online version contains supplementary material available at 10.1186/s12909-024-05365-7.

## Background

Artificial intelligence (AI) has great potential to revolutionize the field of medical education from curricular conception to assessment [1]. AIs used in medical education are mostly generative AI large language models that were developed and validated based on billions to trillions of parameters [2]. AIs hold promise in the incorporation of history-taking, assessment, diagnosis, and management of various disorders [3]. While applications of AIs in undergraduate medical training are being explored, huge ethical challenges remain in terms of data collection, maintaining anonymity, consent, and ownership of the provided data [4]. AIs hold a promising role amongst learners because they can deliver a personalized learning experience by tracking their progress and providing real-time feedback, thereby enhancing their understanding in the areas they are finding difficult [5]. Consequently, a recent survey has shown that medical students have expressed their interest in acquiring competencies related to the use of AIs in healthcare during their undergraduate medical training [6].

Pharmacology and Therapeutics (P & T) is a core discipline embedded in the undergraduate medical curriculum, mostly in the pre-clerkship phase. However, the application of therapeutic principles forms one of the key learning objectives during the clerkship phase of the undergraduate medical career. Student assessment in pharmacology & therapeutics (P&T) is with test items such as multiple-choice questions (MCQs), integrated case cluster questions, short answer questions (SAQs), and objective structured practical examination (OSPE) in the undergraduate medical curriculum. It has been argued that AIs possess the ability to communicate an idea more creatively than humans [7]. It is imperative that with access to billions of trillions of datasets the AI platforms hold promise in playing a crucial role in the conception of various test items related to any of the disciplines in the undergraduate medical curriculum. Additionally, AIs provide an optimized curriculum for a program/course/topic addressing multidimensional problems [8], although robust evidence for this claim is lacking.

The existing literature has evaluated the knowledge, attitude, and perceptions of adopting AI in medical education. Integration of AIs in medical education is the need of the hour in all health professional education. However, the academic medical fraternity facing challenges in the incorporation of AIs in the medical curriculum due to factors such as inadequate grounding in data analytics, lack of high-quality firm evidence favoring the utility of AIs in medical education, and lack of funding [9]. Open-access AI platforms are available free to users without any restrictions. Hence, as a proof-of-concept, we chose to explore the utility of three AI platforms to identify specific learning objectives (SLOs) related to pharmacology discipline in the management of hypertension for medical students at different stages of their medical training.

## Methods

### Study design and ethics

The present study is observational, cross-sectional in design, conducted in the Department of Pharmacology & Therapeutics, College of Medicine and Medical Sciences, Arabian Gulf University, Kingdom of Bahrain, between April and August 2023. Ethical Committee approval was not sought given the nature of this study that neither had any interaction with humans, nor collection of any personal data was involved.

### Study procedure

We conducted the present study in May-June 2023 with the Poe© chatbot interface created by Quora© that provides access to the following three AI platforms:

- Sage Poe [10]: A generative AI search engine developed by Anthropic^©^ that conceives a response based on the written input provided. Quora has renamed Sage Poe as Assistant^©^ from July 2023 onwards.
- Claude-Instant [11]: A retrieval-based AI search engine developed by Anthropic^©^ that collates a response based on pre-written responses amongst the existing databases.
- ChatGPT version 3.5 [12]: A generative architecture-based AI search engine developed by OpenAI^©^ trained on large and diverse datasets.

We queried the chatbots to generate SLOs, A-type MCQs, integrated case cluster MCQs, integrated SAQs, and OSPE test items in the domain of systemic hypertension related to the P&T discipline. Separate prompts were used to generate outputs for pre-clerkship (preclinical) phase students, and at the time of graduation (before starting residency programs). Additionally, we have also evaluated the ability of these AI platforms to estimate the proportion of students correctly answering these test items. We used the following queries for each of these objectives:

#### Specific learning objectives

I. Can you generate specific learning objectives in the pharmacology discipline relevant to undergraduate medical students during their pre-clerkship phase related to anti-hypertensive drugs?
II. Can you generate specific learning objectives in the pharmacology discipline relevant to undergraduate medical students at the time of graduation related to anti-hypertensive drugs?

#### A-type MCQs

In the initial query used for A-type of item, we specified the domains (such as the mechanism of action, pharmacokinetics, adverse reactions, and indications) so that a sample of test items generated without any theme-related clutter, shown below:

I. Write 20 single best answer MCQs with 5 choices related to anti-hypertensive drugs for undergraduate medical students during the pre-clerkship phase of which 5 MCQs should be related to mechanism of action, 5 MCQs related to pharmacokinetics, 5 MCQs related to adverse reactions, and 5 MCQs should be related to indications.

The MCQs generated with the above search query were not based on clinical vignettes. We queried again to generate MCQs using clinical vignettes specifically because most medical schools have adopted problem-based learning (PBL) in their medical curriculum.

II. Write 20 single best answer MCQs with 5 choices related to anti-hypertensive drugs for undergraduate medical students during the pre-clerkship phase using a clinical vignette for each MCQ of which 5 MCQs should be related to the mechanism of action, 5 MCQs related to pharmacokinetics, 5 MCQs related to adverse reactions, and 5 MCQs should be related to indications.

We attempted to explore whether AI platforms can provide useful guidance on standard-setting. Hence, we used the following search query.

III. Can you do a simulation with 100 undergraduate medical students to take the above questions and let me know what percentage of students got each MCQ correct?

#### Integrated case cluster MCQs

I. Write 20 integrated case cluster MCQs with 2 questions in each cluster with 5 choices for undergraduate medical students during the pre-clerkship phase integrating pharmacology and physiology related to systemic hypertension with a case vignette.
II. Write 20 integrated case cluster MCQs with 2 questions in each cluster with 5 choices for undergraduate medical students during the pre-clerkship phase integrating pharmacology and physiology related to systemic hypertension with a case vignette. Please do not include ‘none of the above’ as the choice. (This modified search query was used because test items with ‘None of the above’ option were generated with the previous search query).
III. Write 20 integrated case cluster MCQs with 2 questions in each cluster with 5 choices for undergraduate medical students at the time of graduation integrating pharmacology and physiology related to systemic hypertension with a case vignette.

#### Integrated short answer questions

I. Write a short answer question scenario with difficult questions based on the theme of a newly diagnosed hypertensive patient for undergraduate medical students with the main objectives related to the physiology of blood pressure regulation, risk factors for systemic hypertension, pathophysiology of systemic hypertension, pathological changes in the systemic blood vessels in hypertension, pharmacological management, and non-pharmacological treatment of systemic hypertension.
II. Write a short answer question scenario with moderately difficult questions based on the theme of a newly diagnosed hypertensive patient for undergraduate medical students with the main objectives related to the physiology of blood pressure regulation, risk factors for systemic hypertension, pathophysiology of systemic hypertension, pathological changes in the systemic blood vessels in hypertension, pharmacological management, and non-pharmacological treatment of systemic hypertension.
III. Write a short answer question scenario with questions based on the theme of a newly diagnosed hypertensive patient for undergraduate medical students at the time of graduation with the main objectives related to the physiology of blood pressure regulation, risk factors for systemic hypertension, pathophysiology of systemic hypertension, pathological changes in the systemic blood vessels in hypertension, pharmacological management, and non-pharmacological treatment of systemic hypertension.

#### OSPEs

I. Can you generate 5 OSPE pharmacology and therapeutics prescription writing exercises for the assessment of undergraduate medical students at the time of graduation related to anti-hypertensive drugs?
II. Can you generate 5 OSPE pharmacology and therapeutics prescription writing exercises containing appropriate instructions for the patients for the assessment of undergraduate medical students during their pre-clerkship phase related to anti-hypertensive drugs?
III. Can you generate 5 OSPE pharmacology and therapeutics prescription writing exercises containing appropriate instructions for the patients for the assessment of undergraduate medical students at the time of graduation related to anti-hypertensive drugs?

Both authors independently evaluated the AI-generated outputs, and a consensus was reached. We cross-checked the veracity of answers suggested by AIs as per the Joint National Commission Guidelines (JNC-8) and Goodman and Gilman’s The Pharmacological Basis of Therapeutics (2023), a reference textbook [13, 14]. Errors in the A-type MCQs were categorized as item construction defects, multiple correct answers, and uncertain appropriateness to the learner’s level. Test items in the integrated case cluster MCQs, SAQs and OSPEs were evaluated with the Preliminary Conceptual Framework for Establishing Content Validity of AI-Generated Test Items based on the following domains: technical accuracy, comprehensiveness, education level, and lack of construction defects (Table 1). The responses were categorized as complete and deficient for each domain.

**Table 1:** Preliminary conceptual framework for establishing content validity of AI-generated test items

Domains	Items assessed
Technical	• Are the test items/explanation technically accurate and free from empirical or clinical mistakes?
Comprehensiveness	• Do the test items /explanations sufficiently address relevant topics/subtopics? • Is the within-topic variation (range of examples, patient characteristics, scenario descriptions) at the desired level?
Education level	• Are the test items /model answers appropriate to the education level of the learner? • Is the structure of the questions/explanations aligned to learning outcomes (as per Bloom’s taxonomy)?
Free of construction defects	• Are the test items /answers framed in a way to present a clear “best response” with appropriate and unambiguous distractors? • Does the test item/explanation avoid therapeutic controversies. • Are the test items integrated with the case vignette (without being standalone)?

## Results

### Specific learning objectives

The pre-clerkship phase SLOs identified by Sage Poe, Claude-Instant, and ChatGPT are listed in the electronic supplementary materials 1–3, respectively. In general, a broad homology in SLOs generated by the three AI platforms was observed. All AI platforms identified appropriate action verbs as per Bloom’s taxonomy to state the SLO; action verbs such as describe, explain, recognize, discuss, identify, recommend, and interpret are used to state the learning outcome. The specific, measurable, achievable, relevant, time-bound (SMART) SLOs generated by each AI platform slightly varied. All key domains of antihypertensive pharmacology to be achieved during the pre-clerkship (pre-clinical) years were relevant for graduating doctors. The SLOs addressed current JNC Treatment Guidelines recommended classes of antihypertensive drugs, the mechanism of action, pharmacokinetics, adverse effects, indications/contraindications, dosage adjustments, monitoring therapy, and principles of monotherapy and combination therapy.

The SLOs to be achieved by undergraduate medical students at the time of graduation identified by Sage Poe, Claude-Instant, and ChatGPT listed in electronic supplementary materials 4–6, respectively. The identified SLOs emphasize the application of pharmacology knowledge within a clinical context, focusing on competencies needed to function independently in early residency stages. These SLOs go beyond knowledge recall and mechanisms of action to encompass competencies related to clinical problem-solving, rational prescribing, and holistic patient management. The SLOs generated require higher cognitive ability of the learner: action verbs such as demonstrate, apply, evaluate, analyze, develop, justify, recommend, interpret, manage, adjust, educate, refer, design, initiate & titrate were frequently used.

### A-type MCQs

The MCQs for the pre-clerkship phase identified by Sage Poe, Claude-Instant, and ChatGPT listed in the electronic supplementary materials 7–9, respectively, and those identified with the search query based on the clinical vignette in electronic supplementary materials (10–12).

All MCQs generated by the AIs in each of the four domains specified [mechanism of action (MOA); pharmacokinetics; adverse drug reactions (ADRs), and indications for antihypertensive drugs] are quality test items with potential content validity. The test items on MOA generated by Sage Poe included themes such as renin-angiotensin-aldosterone (RAAS) system, beta-adrenergic blockers (BB), calcium channel blockers (CCB), potassium channel openers, and centrally acting antihypertensives; on pharmacokinetics included high oral bioavailability/metabolism in liver [angiotensin receptor blocker (ARB)-losartan], long half-life and renal elimination [angiotensin converting enzyme inhibitors (ACEI)-lisinopril], metabolism by both liver and kidney (beta-blocker (BB)-metoprolol], rapid onset- short duration of action (direct vasodilator-hydralazine), and long-acting transdermal drug delivery (centrally acting-clonidine). Regarding the ADR theme, dry cough, angioedema, and hyperkalemia by ACEIs in susceptible patients, reflex tachycardia by CCB/amlodipine, and orthostatic hypotension by CCB/verapamil addressed. Clinical indications included the drug of choice for hypertensive patients with concomitant comorbidity such as diabetics (ACEI-lisinopril), heart failure and low ejection fraction (BB-carvedilol), hypertensive urgency/emergency (alpha cum beta receptor blocker-labetalol), stroke in patients with history recurrent stroke or transient ischemic attack (ARB-losartan), and preeclampsia (methyldopa).

Almost similar themes under each domain were identified by the Claude-Instant AI platform with few notable exceptions: hydrochlorothiazide (instead of clonidine) in MOA and pharmacokinetics domains, respectively; under the ADR domain ankle edema/ amlodipine, sexual dysfunction and fatigue in male due to alpha-1 receptor blocker; under clinical indications the best initial monotherapy for clinical scenarios such as a 55-year old male with Stage-2 hypertension; a 75-year-old man Stage 1 hypertension; a 35-year-old man with Stage I hypertension working on night shifts; and a 40-year-old man with stage 1 hypertension and hyperlipidemia.

As with Claude-Instant AI, ChatGPT-generated test items on MOA were mostly similar. However, under the pharmacokinetic domain, immediate- and extended-release metoprolol, the effect of food to enhance the oral bioavailability of ramipril, and the highest oral bioavailability of amlodipine compared to other commonly used antihypertensives were the themes identified. Whereas the other ADR themes remained similar, constipation due to verapamil was a new theme addressed. Notably, in this test item, amlodipine was an option that increased the difficulty of this test item because amlodipine therapy is also associated with constipation, albeit to a lesser extent, compared to verapamil. In the clinical indication domain, the case description asking *“most commonly used in the treatment of hypertension and heart failure”* is controversial because the options listed included losartan, ramipril, and hydrochlorothiazide but the suggested correct answer was ramipril. This is a good example to stress the importance of vetting the AI-generated MCQ by experts for content validity and to assure robust psychometrics. The MCQ on the most used drug in the treatment of *“hypertension and diabetic nephropathy”* is more explicit as opposed to *“hypertension and diabetes”* by Claude-Instant because the therapeutic concept of reducing or delaying nephropathy must be distinguished from prevention of nephropathy, although either an ACEI or ARB is the drug of choice for both indications.

It is important to align student assessment to the curriculum; in the PBL curriculum, MCQs with a clinical vignette are preferred. The modification of the query specifying the search to generate MCQs with a clinical vignette on domains specified previously gave appropriate output by all three AI platforms evaluated (Sage Poe; Claude- Instant; Chat GPT). The scenarios generated had a good clinical fidelity and educational fit for the pre-clerkship student perspective.

The errors observed with AI outputs on the A-type MCQs are summarized in Table 2. No significant pattern was observed except that Claude-Instant© generated test items in a stereotyped format such as the same choices for all test items related to pharmacokinetics and indications, and all the test items in the ADR domain are linked to the mechanisms of action of drugs. This illustrates the importance of reviewing AI-generated test items by content experts for content validity to ensure alignment with evidence-based medicine and up-to-date treatment guidelines.

**Table 2:** Comparison of types of errors in the A-type MCQs between the AI platforms in pre-clerkship phase and at graduation

Types of errors	Sage Poe©		Chart GPT©		Claude-Instant©	
	Pre-clerkship (*n* = 5)	At graduation (*n* = 5)	Pre-clerkship (*n* = 5)	At graduation (*n* = 5)	Pre-clerkship (*n* = 5)	At graduation (*n* = 5)
**Pharmacokinetics**						
Item construction defect	5^b, c, d, e, f^	4 ^ac, ad, ae, ag^	None	None	5 ^au^	None
More than one correct option	None	1^s^	1^s^	None	None	None
Appropriateness to learners’ level controversial	3 ^b, c, e^	1^ac^	2 ^r, t^	1^an^	None	None
**Mechanisms of action**						
Item construction defect	None	None	2 ^q, u^	1^aL^	None	None
More than one correct option	1^a^	1^ab^	None	3^am, ao, ap^	None	None
Appropriateness to learners’ level controversial	None	None	1^v^	None	None	None
**Adverse drug reactions**						
Item construction defect	None	None	None	None	None	None
More than one correct option	1^g^	1^ah^	1^z^	2^aq, ar^	None	None
Appropriateness to learners’ level controversial	1^h^	None	None	None	None	None
**Indications**						
Item construction defect	None	None	1^z^	None	1 ^p^	None
More than one correct option	3 ^i, k, l^	2 ^a.i., ak^	2 ^y, aa^	1^as^	4 ^m, n, o, p^	None
Appropriateness to learners’ level controversial	2 ^j, k^	1^aj^	None	None	1 ^n^	None

a-lisinopril (test item #1); b-bioavailability and metabolized by liver (item #6); c-renal elimination and long half-life (item# 7); d-metabolized by both liver and kidney (item #8); e-rapid onset of action and short duration (item #9); f- transdermal patch and duration of action (item #10); g-lisinopril and losartan (item #13); h-verapamil and orthostatic hypotension (item #15); i-lisinopril and losartan (item # 16); j-carvedilol for heart failure with reduced ejection fraction (item #17); k-lisinopril and losartan for stroke prevention (item #18); L-hydralazine and methyldopa (item #20); m-lisinopril and losartan (item #17); n-HT Rx in night shift worker (item #18); o- treatment of hypertension and chronic kidney disease (item #19); p- treatment of hypertension and hyperlipidemia (item #20); q-metoprolol and its effects on blood vessels (item #4); R-longest half-life among antihypertensives (item # 6); s- four possible correct answers (item #7); t-immediate and extended-release preparations (item # 8); u- antihypertensive drug to be taken with food (item #9); v-highest bioavailable antihypertensive drug (item #10); x-losartan and ramipril cause hyperkalemia (item#11); y-losartan and ramipril can be used for treating hypertension and heart failure (item #16); z- hypertension with angina and amlodipine (item #17); aa-losartan and ramipril in hypertension and diabetic Nephropathy (item #18); ab-lisinopril and losartan (item #4); ac-high bioavailability and metabolized by liver (item #6); ad-eliminated by kidneys and long half-life (item #7); ae-metoprolol metabolized by both liver and kidneys (item #8); af-rapid onset and short duration (item #9); ag-transdermal patch and longer duration (item #10); ah-lisinopril and losartan (item #13); ai-hydrochlorothiazide, lisinopril and losartan are indicated for hypertension and diabetes mellitus (item #16); aj-losartan as antihypertensive drug for preventing recurrent stroke (item #19); ak-methyldopa and hydralazine can be used in preeclampsia (item #20); al-ramipril inhibits RAAS (item #2); am-losartan and candesartan blocks type II receptors (item #3); an-doxazosin with longest duration of action (item #6); ao-ramipril and losartan contra-indicated in renal impairment (item #8); ap-losartan, ramipril and spironolactone cause hyperkalemia (item # 13); ar- hydrochlorothiazide and hydrochlorothiazide/triamterene can result in photosensitivity (item # 15); as-losartan and ramipril reduce morbidity and mortality in heart failure (item #16); and au-The question should be what type of pharmacokinetic characteristics best describes the respective drug (items #6 to #10)

The test items generated by ChatGPT had the advantage of explanations supplied rendering these more useful for learners to support self-study. The following examples illustrate this assertion: “*A patient with hypertension is started on a medication that works by blocking beta-1 receptors in the heart* (metoprolol)”. Metoprolol is a beta blocker that works by blocking beta-1 receptors in the heart, which reduces heart rate and cardiac output, resulting in a decrease in blood pressure. However, this explanation is incomplete because there is no mention of other less important mechanisms, of beta receptor blockers on renin release. Also, these MCQs were mostly recall type: *Which of the following medications is known to have a significant first-pass effect?* The explanation reads: propranolol is known to have a significant first pass-effect, meaning that a large portion of the drug is metabolized by the liver before it reaches systemic circulation. Losartan, amlodipine, ramipril, and hydrochlorothiazide do not have significant first-pass effect. However, it is also important to extend the explanation further by stating that the first-pass effect of propranolol does not lead to total loss of pharmacological activity because the metabolite hydroxy propranolol also has potent beta-blocking activity. Another MCQ test item had a construction defect: *“A patient with hypertension is started on a medication that can cause photosensitivity. Which of the following medications is most likely responsible?”* Options included: losartan, amlodipine, ramipril, hydrochlorothiazide, hydrochlorothiazide/triamterene. The explanation states that hydrochlorothiazide (HCTZ) can cause photosensitivity, which is increased sensitivity to sunlight that can cause skin rashes and sunburns. Losartan, amlodipine, ramipril, and HCTZ/triamterene are less likely to cause photosensitivity. However, it is irrational to claim that HCTZ/triamterene, one of the wrong options, is less likely to cause photosensitivity. The difficulty index of such test items is likely unacceptable in high-stake tests to make equitable psychometric decisions.

The responses from Sage Poe, Claude-Instant, and ChatGPT on guidance for standard-setting methods are listed in the electronic supplementary materials 13–15. Sage Poe offered this information with the caveat that *“the results of this simulation were purely hypothetical and do not reflect the actual performance of any real students”.* The simulation estimates of examinees correctly answering test items ranged from 60 to 90% for the mechanism of action, 65–85% for pharmacokinetics, 60–80% for adverse reactions, and 75–90% for clinical indications of antihypertensives.

In contrast, Claude-Instant admitted that “Performing a full simulation with 100 medical students is beyond the scope of what I can do as an AI assistant. I can generate sample MCQ questions and answers, but I do not have access to a population of medical students to administer the questions. A true simulation that administers questions to real students and analyzes the results would require access to a database of medical students, a platform to deliver the questions, and code to analyze the responses. As an AI assistant, I lack these capabilities. While I cannot perform a full simulation, here are some considerations for administering MCQ questions to medical students: provide details on the purpose and instructions for the test to ensure students understand what is expected out of them; consider including some easier questions in addition to more challenging ones to boost students’ confidence and motivation; avoid ambiguous wording and ensure each question has only one unambiguously correct answer option; when analyzing results, look for questions factors that may have impacted performance, like unclear wording or answer options that are too similar. Focusing on weak questions can help improve future tests; compare performance across different student groups to identify areas where more teaching may be needed; provide detailed answer explanations and review sessions after the test to help students learn from any errors and reinforce correct concepts”. However, ChatGPT offered the simulation result estimates for the 20 MCQ on antihypertensive drugs: mechanism of action ranged from 67 to 92%, pharmacokinetics 63–86%, adverse effects 65–82%, and clinical indications 64–89%. Furthermore, it also stated that “Overall, the performance of the students was quite good, with most questions having a response rate of over 70%. However, there were some questions where the projected correct response rate was rather low, such as Question #5 (Mechanism of Action of Hydrochlorothiazide; see Electronic Supplementary Material 12) and Question 18 (Indications for Verapamil; see Electronic Supplementary Material 10). This may suggest areas where students need more focused education or review.”

### Integrated case cluster MCQs

We asked AI assistants to generate 20 integrated case cluster MCQs with 2 test items in each cluster with five options for undergraduate medical students in the pre-clerkship phase integrating pharmacology and physiology related to systemic hypertension with a case vignette and the responses by Sage Poe, Claude-Instant, and ChatGPT are listed in the electronic supplementary materials (16–18). In all instances, the test items generated had focused case descriptions in the form of a clinical vignette, and horizontal integration across the pathophysiology of hypertension and pharmacology of antihypertensive drugs. These test items mostly targeted the ‘knows (knowledge)’ or ‘knows how (competence)’ level on Miller’s pyramid and are suitable for assessing the clinical competence of pre-clerkship medical students, especially in an integrated PBL curriculum. Both the AI assistants generated excellent clinical vignettes and themes; however, most of the cluster MCQs by ChatGPT had *“None of the above”* as an option, which is often considered a test item construction flaw. Notwithstanding these limitations, case cluster integrated test items are valuable for learners to integrate their knowledge of different basic medical sciences and their application to clinical sciences. This integrated approach can be used for both instructional and student assessment purposes to make the course more meaningful. Indeed, one of the basic tenets of PBL is curriculum integration.

In the next query, we asked AI assistants to write integrated case cluster MCQs with 2 test items in each cluster with 5 options for undergraduate medical students at the time of graduation integrating pharmacology and physiology of hypertension with a case vignette. The responses are listed in the electronic supplementary materials 19–21. Claude-Instant has provided only partial responses to both the queries. Overall, the information provided in case vignettes requires the learner to interpret the clinical information and make therapeutic decisions. Such test items are appropriate for assessing the minimum competency of medical school graduates to start internship or residency programs; these MCQs are generally comparable to test items used in USMLE Part 2. The evaluation of the integrated case cluster test items provided by all the AI platforms is summarized in Table 3. In general, the AI outputs had at least one construction defect, and did not include all the core sub-topics to a sufficient degree.

**Table 3:** Assessment of test items using the preliminary conceptual framework for establishing content validity of AI-generated test items (integrated case clusters, SAQs and OSPEs)

Types of errors	Sage Poe©		Chart GPT©		Claude-Instant©	
	Pre-clerkship	At graduation	Pre-clerkship	At graduation	Pre-clerkship	At graduation
**Integrated case cluster**						
Technical accuracy	Complete	Complete	Complete	Complete	Deficient	Complete ^a^
Comprehensiveness	Deficient	Deficient	Deficient	Deficient	Deficient	Deficient ^a^
Education level	Complete	Deficient	Deficient	Deficient	Complete	Complete ^a^
Free of construction defects	Deficient	Deficient	Deficient	Deficient	Deficient	Complete ^a^
**Short answer questions**						
Technical accuracy	Complete	Complete	Complete	Complete	Complete	Complete
Comprehensiveness	Complete	Complete	Complete	Complete	Complete	Complete
Education level	Complete	Deficient	Complete	Deficient	Complete	Complete
Free of construction defects	Deficient	Deficient	Deficient	Deficient	Deficient	Complete
**OSPEs**						
Technical accuracy	Not Available		Complete	Complete	Complete	Complete
Comprehensiveness			Complete	Complete	Complete	Complete
Education level			Complete	Complete	Complete	Complete
Free of construction defects			Complete	Complete	Complete	Complete

a- Only a small portion of the requested test items were provided by the concerned AI tool

When asked to simulate the above-generated test items to 100 undergraduate medical students at the time of graduation and provide a percentage of students who are likely to correctly answer each item, the AI assistant responded:

“*As an AI language model, I cannot simulate the responses of 100 undergraduate medical students likely to obtain the correct answer for each question. However, I can provide some general information about the difficulty level of each question based on the complexity of the concepts being tested and the level of integration between pharmacology and physiology*”.

AI assistant went on to clarify that questions that require knowledge of basic pharmacology principles, such as the mechanism of action of specific drugs, are likely to be easier for students to answer correctly. Test items that require an understanding of the physiological mechanisms underlying hypertension and correlating with symptoms are likely to be more challenging for students. The AI assistant sorted these test items into two categories accordingly. Overall, the difficulty level of the test item is based on the level of integration between pharmacology and pathophysiology. Test items that require an understanding of both pharmacological and physiological mechanisms are likely to be more challenging for students requiring a strong foundation in both pharmacology and physiology concepts to be able to correctly answer integrated case-cluster MCQs.

### Short answer questions

The responses to a search query on generating SAQs appropriate to the pre-clerkship phase Sage Poe, Claude-Instant, and ChatGPT generated items are listed in the electronic supplementary materials 22–24 for difficult questions and 25–27 for moderately difficult questions.

It is apparent from these case vignette descriptions that the short answer question format varied. Accordingly, the scope for asking individual questions for each scenario is open-ended. In all instances, model answers are supplied which are helpful for the course instructor to plan classroom lessons, identify appropriate instructional methods, and establish rubrics for grading the answer scripts, and as a study guide for students.

We then wanted to see to what extent AI can differentiate the difficulty of the SAQ by replacing the search term “difficult” with “moderately difficult” in the above search prompt: the changes in the revised case scenarios are substantial. Perhaps the context of learning and practice (and the level of the student in the MD/medical program) may determine the difficulty level of SAQ generated. It is worth noting that on changing the search from cardiology to internal medicine rotation in Sage Poe the case description also changed. Thus, it is essential to select an appropriate AI assistant, perhaps by trial and error, to generate quality SAQs. Most of the individual questions tested stand-alone knowledge and did not require students to demonstrate integration.

The responses of Sage Poe, Claude-Instant, and ChatGPT for the search query to generate SAQs at the time of graduation are listed in the electronic supplementary materials 28–30. It is interesting to note how AI assistants considered the stage of the learner while generating the SAQ. The response by Sage Poe is illustrative for comparison. *“You are a newly graduated medical student who is working in a hospital”* versus *“You are a medical student in your pre-clerkship.”*

Some questions were retained, deleted, or modified to align with competency appropriate to the context (Electronic Supplementary Materials 28–30). Overall, the test items at both levels from all AI platforms were technically accurate and thorough addressing the topics related to different disciplines (Table 3). The differences in learning objective transition are summarized in Table 4. A comparison of learning objectives revealed that almost all objectives remained the same except for a few (Table 5).

**Table 4:** Comparison of the SAQ test items generated by Sage Poe for pre-clerkship phase and graduating students

Pre-clerkship phase	At graduation
Physiological mechanisms that regulate blood pressure in the body.	Pathophysiology of systemic hypertension.
Risk factors for systemic hypertension.	Potential complications of untreated hypertension.
Diuretics to lower blood pressure.	ACEI to lower blood pressure.
Frequency of checking blood pressure in patients with hypertension.	Recommended blood pressure target for patients with hypertension.

ACEI– Angiotensin-converting enzyme inhibitors

**Table 5:** Comparison of learning objectives in SAQ generated for pre-clerkship phase and graduating students

Pre-clerkship phase	At graduation
Mechanism of action of diuretics in hypertension treatment.	Mechanism of action of ACEI in hypertension treatment.
Frequency of blood pressure checks for patients with hypertension.	Blood pressure targets for patients with hypertension.

ACEI– Angiotensin-converting enzyme inhibitors

A similar trend was apparent with test items generated by other AI assistants, such as ChatGPT. The contrasting differences in questions are illustrated by the vertical integration of basic sciences and clinical sciences (Table 6).

**Table 6:** Comparison of the SAQ test items generated by ChatGPT for pre-clerkship phase and graduating students

Pre-clerkship phase	At graduation
How does sympathetic nervous system activation affect blood pressure?	How does sympathetic nervous system activation affect blood pressure, and how can medications targeting this system be used to manage hypertension?
What are pathological changes that occur in the systemic blood vessels in hypertension?	What are pathological changes that occur in the systemic blood vessels in hypertension, and how do these changes contribute to cardiovascular complications?
What are non-pharmacological treatment options in hypertension?	What are non-pharmacological treatment options in hypertension, and how effective are they in managing this condition?
What are the pharmacological treatment options for hypertension?	What are the pharmacological treatment options for hypertension, and how do they work?

Taken together, these in-depth qualitative comparisons suggest that AI assistants such as Sage Poe and ChatGPT consider the learner’s stage of training in designing test items, learning outcomes, and answers expected from the examinee. It is critical to state the search query explicitly to generate quality output by AI assistants.

### OSPEs

The OSPE test items generated by Claude-Instant and ChatGPT appropriate to the pre-clerkship phase (without mentioning “appropriate instructions for the patients”) are listed in the electronic supplementary materials 31 and 32 and with patient instructions on the electronic supplementary materials 33 and 34. For reasons unknown, Sage Poe did not provide any response to this search query.

The five OSPE items generated were suitable to assess the prescription writing competency of pre-clerkship medical students. The clinical scenarios identified by the three AI platforms were comparable; these scenarios include patients with hypertension and impaired glucose tolerance in a 65-year-old male, hypertension with chronic kidney disease (CKD) in a 55-year-old woman, resistant hypertension with obstructive sleep apnea in a 45-year-old man, and gestational hypertension at 32 weeks in a 35-year-old (Claude-Instant AI). Incorporating appropriate instructions facilitates the learner’s ability to educate patients and maximize safe and effective therapy. The OSPE item required students to write a prescription with guidance to start conservatively, choose an appropriate antihypertensive drug class (drug) based on the patients’ profile, specifying drug name, dose, dosing frequency, drug quantity to be dispensed, patient name, date, refill, and caution as appropriate, in addition to prescribers’ name, signature, and license number. In contrast, ChatGPT identified clinical scenarios to include patients with hypertension and CKD, hypertension and bronchial asthma, gestational diabetes, hypertension and heart failure, and hypertension and gout (ChatGPT). Guidance for dosage titration, warnings to be aware, safety monitoring, and frequency of follow-up and dose adjustment. These test items are designed to assess learners’ knowledge of P & T of antihypertensives, as well as their ability to provide appropriate instructions to patients. These clinical scenarios for writing prescriptions assess students’ ability to choose an appropriate drug class, write prescriptions with proper labeling and dosing, reflect drug safety profiles, and risk factors, and make modifications to meet the requirements of special populations. The prescription is required to state the drug name, dose, dosing frequency, patient name, date, refills, and cautions or instructions as needed. A conservative starting dose, once or twice daily dosing frequency based on the drug, and instructions to titrate the dose slowly if required.

The responses from Claude-Instant and ChatGPT for the search query related to generating OSPE test items at the time of graduation are listed in electronic supplementary materials 35 and 36. In contrast to the pre-clerkship phase, OSPEs generated for graduating doctors’ competence assessed more advanced drug therapy comprehension. For example, writing a prescription for:

(1) A 65-year- old male with resistant hypertension and CKD stage 3 to optimize antihypertensive regimen required the answer to include starting ACEI and diuretic, titrating the dosage over two weeks, considering adding spironolactone or substituting ACEI with an ARB, and need to closely monitor serum electrolytes and kidney function closely.

(2) A 55-year-old woman with hypertension and paroxysmal arrhythmia required the answer to include switching ACEI to ARB due to cough, adding a CCB or beta blocker for rate control needs, and adjusting the dosage slowly and monitoring for side effects.

(3) A 45-year-old man with masked hypertension and obstructive sleep apnea require adding a centrally acting antihypertensive at bedtime and increasing dosage as needed based on home blood pressure monitoring and refer to CPAP if not already using one.

(4) A 75-year-old woman with isolated systolic hypertension and autonomic dysfunction to require stopping diuretic and switching to an alpha blocker, upward dosage adjustment and combining with other antihypertensives as needed based on postural blood pressure changes and symptoms.

(5) A 35-year-old pregnant woman with preeclampsia at 29 weeks require doubling methyldopa dose and consider adding labetalol or nifedipine based on severity and educate on signs of worsening and to follow-up immediately for any concerning symptoms.

These case scenarios are designed to assess the ability of the learner to comprehend the complexity of antihypertensive regimens, make evidence-based regimen adjustments, prescribe multidrug combinations based on therapeutic response and tolerability, monitor complex patients for complications, and educate patients about warning signs and follow-up.

A similar output was provided by ChatGPT, with clinical scenarios such as prescribing for patients with hypertension and myocardial infarction; hypertension and chronic obstructive pulmonary airway disease (COPD); hypertension and a history of angina; hypertension and a history of stroke, and hypertension and advanced renal failure. In these cases, wherever appropriate, pharmacotherapeutic issues like taking ramipril after food to reduce side effects such as giddiness; selection of the most appropriate beta-blocker such as nebivolol in patients with COPD comorbidity; the importance of taking amlodipine at the same time every day with or without food; preference for telmisartan among other ARBs in stroke; choosing furosemide in patients with hypertension and edema and taking the medication with food to reduce the risk of gastrointestinal adverse effect are stressed.

The AI outputs on OSPE test times were observed to be technically accurate, thorough in addressing core sub-topics suitable for the learner’s level and did not have any construction defects (Table 3). Both AIs provided the model answers with explanatory notes. This facilitates the use of such OSPEs for self-assessment by learners for formative assessment purposes. The detailed instructions are helpful in creating optimized therapy regimens, and designing evidence-based regimens, to provide appropriate instructions to patients with complex medical histories. One can rely on multiple AI sources to identify, shortlist required case scenarios, and OSPE items, and seek guidance on expected model answers with explanations. The model answer guidance for antihypertensive drug classes is more appropriate (rather than a specific drug of a given class) from a teaching/learning perspective. We believe that these scenarios can be refined further by providing a focused case history along with relevant clinical and laboratory data to enhance clinical fidelity and bring a closer fit to the competency framework.

## Discussion

In the present study, AI tools have generated SLOs that comply with the current principles of medical education [15]. AI tools are valuable in constructing SLOs and so are especially useful for medical fraternities where training in medical education is perceived as inadequate, more so in the early stages of their academic career. Data suggests that only a third of academics in medical schools have formal training in medical education [16] which is a limitation. Thus, the credibility of alternatives, such as the AIs, is evaluated to generate appropriate course learning outcomes.

We observed that the AI platforms in the present study generated quality test items suitable for different types of assessment purposes. The AI-generated outputs were similar with minor variation. We have used generative AIs in the present study that could generate new content from their training dataset [17]. Problem-based and interactive learning approaches are referred to as “bottom-up” where learners obtain first-hand experience in solving the cases first and then indulge in discussion with the educators to refine their understanding and critical thinking skills [18]. We suggest that AI tools can be useful for this approach for imparting the core knowledge and skills related to Pharmacology and Therapeutics to undergraduate medical students. A recent scoping review evaluating the barriers to writing quality test items based on 13 studies has concluded that motivation, time constraints, and scheduling were the most common [19]. AI tools can be valuable considering the quick generation of quality test items and time management. However, as observed in the present study, the AI-generated test items nevertheless require scrutiny by faculty members for content validity. Moreover, it is important to train faculty in AI technology-assisted teaching and learning. The General Medical Council recommends taking every opportunity to raise the profile of teaching in medical schools [20]. Hence, both the academic faculty and the institution must consider investing resources in AI training to ensure appropriate use of the technology [21].

The AI outputs assessed in the present study had errors, particularly with A-type MCQs. One notable observation was that often the AI tools were unable to differentiate the differences between ACEIs and ARBs. AI platforms access several structured and unstructured data, in addition to images, audio, and videos. Hence, the AI platforms can commit errors due to extracting details from unauthenticated sources [22] created a framework identifying 28 factors for reconstructing the path of AI failures and for determining corrective actions. This is an area of interest for AI technical experts to explore. Also, this further iterates the need for human examination of test items before using them for assessment purposes.

There are concerns that AIs can memorize and provide answers from their training dataset, which they are not supposed to do [23]. Hence, the use of AIs-generated test items for summative examinations is debatable. It is essential to ensure and enhance the security features of AI tools to reduce or eliminate cross-contamination of test items. Researchers have emphasized that AI tools will only reach their potential if developers and users can access full-text non-PDF formats that help machines comprehend research papers and generate the output [24].

AI platforms may not always have access to all standard treatment guidelines. However, in the present study, it was observed that all three AI platforms generally provided appropriate test items regarding the choice of medications, aligning with recommendations from contemporary guidelines and standard textbooks in pharmacology and therapeutics. The prompts used in the study were specifically focused on the pre-clerkship phase of the undergraduate medical curriculum (and at the time of their graduation) and assessed fundamental core concepts, which were also reflected in the AI outputs. Additionally, the recommended first-line antihypertensive drug classes have been established for several decades, and information regarding their pharmacokinetics, ADRs, and indications is well-documented in the literature.

Different paradigms and learning theories have been proposed to support AI in education. These paradigms include AI- directed (learner as recipient), AI-supported (learner as collaborator), and AI-empowered (learner as leader) that are based on Behaviorism, Cognitive-Social constructivism, and Connectivism-Complex adaptive systems, respectively [25]. AI techniques have potential to stimulate and advance instructional and learning sciences. More recently a three- level model that synthesizes and unifies existing learning theories to model the roles of AIs in promoting learning process has been proposed [26]. The different components of our study rely upon these paradigms and learning theories as the theoretical underpinning.

### Strengths and limitations

To the best of our knowledge, this is the first study evaluating the utility of AI platforms in generating test items related to a discipline in the undergraduate medical curriculum. We have evaluated the AI’s ability to generate outputs related to most types of assessment in the undergraduate medical curriculum. The key lessons learnt for improving the AI-generated test item quality from the present study are outlined in Table 7. We used a structured framework for assessing the content validity of the test items. However, we have demonstrated using a single case study (hypertension) as a pilot experiment. We chose to evaluate anti-hypertensive drugs as it is a core learning objective and one of the most common disorders relevant to undergraduate medical curricula worldwide. It would be interesting to explore the output from AI platforms for other common (and uncommon/region-specific) disorders, non-/semi-core objectives, and disciplines other than Pharmacology and Therapeutics. An area of interest would be to look at the content validity of the test items generated for different curricula (such as problem-based, integrated, case-based, and competency-based) during different stages of the learning process. Also, we did not attempt to evaluate the generation of flowcharts, algorithms, or figures for generating test items. Another potential area for exploring the utility of AIs in medical education would be repeated procedural practices such as the administration of drugs through different routes by trainee residents [27]. Several AI tools have been identified for potential application in enhancing classroom instructions and assessment purposes pending validation in prospective studies [28]. Lastly, we did not administer the AI-generated test items to students and assessed their performance and so could not comment on the validity of test item discrimination and difficulty indices. Additionally, there is a need to confirm the generalizability of the findings to other complex areas in the same discipline as well as in other disciplines that pave way for future studies. The conceptual framework used in the present study for evaluating the AI-generated test items needs to be validated in a larger population. Future studies may also try to evaluate the variations in the AI outputs with repetition of the same queries.

**Table 7:** Key take home messages for improving AI-generated test item quality

Key take home messages
• Compare multiple AI platforms to evaluate the output fidelity.
• Link course syllabus, SLOs, expected competency, and learner’s stage in the program.
• Use unambiguous and specific search prompts to refine the search iteration strategy.
• Decide whether test items sought are for formative or summative purpose.
• Clarify the expected test items match on Bloom’s taxonomy.
• Seek high fidelity clinical vignette to promote context-based learning.
• Define the level of integration appropriate to learner’s stage in the program.
• Integrate the complexity of OSPE clinical scenarios to patient-instructions.
• Recognize the limitations of AIs such as a limited access to all treatment guidelines.
• Ensure the validity of AI generated test items by content experts.
• Evaluate simulation- based standard setting guidance offered by AIs to real world situation.

## Conclusion

Notwithstanding ongoing discussions and controversies, AI tools are potentially useful adjuncts to optimize instructional methods, test blueprinting, test item generation, and guidance for test standard-setting appropriate to learners’ stage in the medical program. However, experts need to critically review the content validity of AI-generated output. These challenges and caveats are to be addressed before the use of widespread use of AIs in medical education can be advocated.

### Electronic supplementary material

Below is the link to the electronic supplementary material.

Supplementary Material 1

## Data Availability

All the data included in this study are provided as Electronic Supplementary Materials.
